# STAT3: A Novel Molecular Mediator of Resistance to Chemoradiotherapy

**DOI:** 10.3390/cancers6041986

**Published:** 2014-09-29

**Authors:** Melanie Spitzner, Reinhard Ebner, Hendrik A. Wolff, B. Michael Ghadimi, Jürgen Wienands, Marian Grade

**Affiliations:** 1 Department of General, Visceral and Pediatric Surgery, University Medicine Göttingen, Robert-Koch-Str. 40, Göttingen 37075, Germany; E-Mail: mghadimi@med.uni-goettingen.de; 2 Genetics Branch, National Cancer Institute, National Institutes of Health, Bethesda, MD 20892, USA; E-Mail: reinhard.ebner@yahoo.com; 3 Department of Radiotherapy and Radiooncology, University Medicine Göttingen, Robert-Koch-Str. 40, Göttingen 37075, Germany; E-Mail: hendrik.wolff@med.uni-goettingen.de; 4 Department of Cellular and Molecular Immunology, University Medicine Göttingen, Humboldtallee 34, Göttingen 37073, Germany; E-Mail: jwienan@uni-goettingen.de

**Keywords:** STAT3, cancer, radiotherapy, chemoradiotherapy, chemoradiotherapy-resistance, chemoradiotherapy-sensitization, molecular target

## Abstract

Chemoradiotherapy (CRT) represents a standard treatment for many human cancers, frequently combined with radical surgical resection. However, a considerable percentage of primary cancers are at least partially resistant to CRT, which represents a substantial clinical problem, because it exposes cancer patients to the potential side effects of both irradiation and chemotherapy. It is therefore exceedingly important to determine the molecular characteristics underlying CRT-resistance and to identify novel molecular targets that can be manipulated to re-sensitize resistant tumors to CRT. In this review, we highlight much of the recent evidence suggesting that the signal transducer and activator of transcription 3 (STAT3) plays a prominent role in mediating CRT-resistance, and we outline why inhibition of STAT3 holds great promise for future multimodal treatment concepts in oncology.

## 1. Introduction

### 1.1. The Clinical Problem of Resistance to Chemoradiotherapy

Radiation therapy (RT) is an integral part of modern multimodal treatment concepts for various tumor entities, because it increases local response and control rates, often facilitates complete surgical resection (tumor-free resection margins) and, thus, is able to improve patient survival [1,2,3,4]. In multimodal treatment strategies, RT is typically combined with surgical approaches. Radiation therapy can be administered as a single-treatment modality or combined with concomitant chemotherapy, which may serve as a radiosensitizer. The goal of chemoradiotherapy (CRT) is to achieve tumor cell damage, primarily achieved through irradiation-mediated effects. These effects are largely the result of DNA damage, which either occurs directly through ionization within the DNA molecule or indirectly from the action of chemical radicals, which are also formed during irradiation [5,6,7].

However, the response to CRT varies tremendously from one patient to another. While there is a clear benefit for patients who experience a pronounced tumor remission after CRT, this poses a particular problem for patients with *a priori* resistant tumors, because they may be exposed to irradiation and chemotherapy, treatment regimens that are expensive and at times toxic. Accordingly, there is a strong clinical need to identify novel molecular targets that can be manipulated in order to sensitize *a priori* resistant tumors to multimodal treatment regimens and to increase the fraction of cancer patients that respond to CRT. A number of recent studies revealed that members of the Signal Transducers and Activators of Transcription (STAT) family of proteins, and most prominently, STAT3, are promising candidates for such drug targets. Here, we summarize the role of STAT3 in various tumor entities and how this knowledge may be used to improve the treatment of CRT-resistant tumors.

### 1.2. The JAK/STAT Signaling Paradigm in Cancer

STAT proteins were discovered more than 20 years ago by James E. Darnell and colleagues when they investigated the molecular mechanisms of how interferons mediate their potent antiviral activities [8,9]. However, it soon became clear that STATs are involved in the regulation of many specialized, as well as housekeeping cell processes. STATs function as essential signal transducing effector proteins of cytokine- or hormone-induced pathways that control the development, proliferation or differentiation, as well as the homeostasis of many cell types [10,11,12,13]. Cell surface receptors that utilize STAT effectors are intracellularly associated with members of the Janus family of protein tyrosine kinases (JAKs) that, upon receptor activation by ligand binding, phosphorylate STAT proteins at specific tyrosine residues. Phosphorylated STATs homo- or hetero-dimerize, by virtue of their Src homology (SH) 2 domain, and subsequently translocate from the cytosol into the nucleus to regulate the transcription of specific target genes [14,15,16,17].

The pathway is regulated at all of its component steps, for example by the action of protein phosphatases, by inhibitors of phosphorylation or by nuclear inhibitory factors. Prominent negative regulators are members of the suppressors of cytokine signaling (SOCS) and protein inhibitors of activated STAT (PIAS) family [18]. Furthermore, STAT proteins may also be activated through direct phosphorylation by some receptor tyrosine kinases, e.g., EGFR, or non-receptor kinases, e.g., c-SRC [16,19,20,21].

The JAK/STAT pathway has been observed in diverse animal species, including slime molds, worms, flies and all vertebrates, but not in fungi or plants. Since the characterization of their first prototype members, seven STATs and four JAKs have been described in mammalian cells. Among the STATs, STAT3 is more highly conserved than others and appears to be less pathway-specific, to be activated by a wider range of factors and stimuli and to possibly modulate a wider variety of processes [22,23]. STAT3 may therefore represent the more primordial, general STAT type.

**Figure 1:**
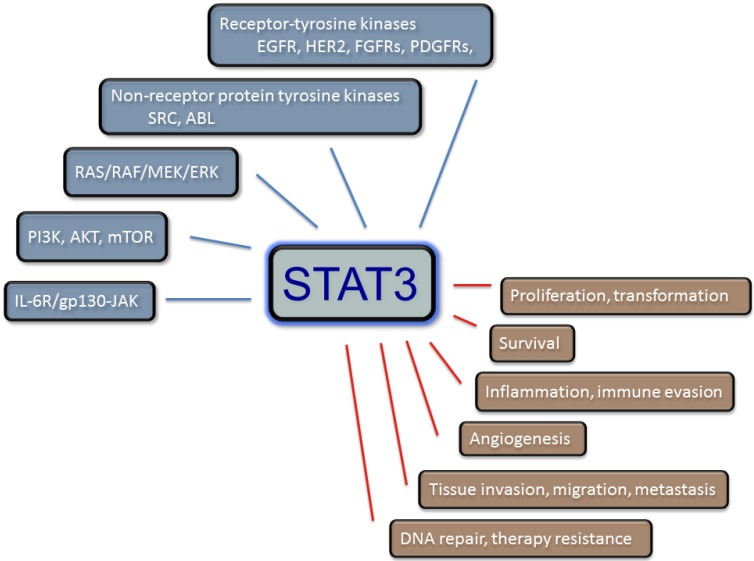
STAT3 modulates several signaling pathways and affects numerous cellular processes.

Concluding from data generated while studying its role in controlling cell cycle progression, apoptosis and tumorigenesis for over a decade, STAT3 has been recognized as a prominent oncogene. It is required to maintain the transformed phenotype in many cells and organs, and many cancer-derived cells depend on its sustained presence and activity [24,25,26,27] (Figure 1).

However, as has been the case with STAT1 some years earlier, a potentially tumor-suppressive role for STAT3 has also been recently proposed [28]. On the other hand, in light of the many different signaling pathways and complex regulatory networks that STAT3 participates in, context-dependent effects cannot be surprising [24,25,29,30].

Sustained activation of STAT3 can promote tumor cell survival and migration. Persistent STAT3 activity can also enhance stem cell-like and mesenchymal properties, making obstruction of the pathway through which it signals a potentially attractive therapeutic strategy. In several studies, increasing in number through the last few years, STAT3 has also been implicated in resistance to radiation. Its combined roles in growth control, cancer stem cell (CSC) maintenance and radioresistance have lent growing credence to the therapy-sensitizing potential of STAT3 inhibition [25,31,32,33,34,35].

## 2. STAT3 as a Molecular Target to Sensitize Tumors to (Chemo-) Radiotherapy

Over the last decade, a large number of tumor-derived cell lines, as well as many primary human cancer tissues have been reported to overexpress and/or constitutively activate STAT3 [36,37]. Conceivably owing to its diverse functions in controlling various (patho-) physiological cellular processes, there is a mounting body of evidence demonstrating that STAT3 also plays a critical role in mediating resistance to CRT.

### 2.1. Brain Tumors

Gliomas are the most frequent and malignant type of brain tumors in adults [38,39]. The standard treatment approach for this tumor type consists of a complete surgical resection followed by CRT and, partly, chemotherapy [38,40].

The first hint that STAT3 may be involved in the aggressiveness of human gliomas was the discovery of an amplified expression of the *Interleukin-6 (IL-6)* gene in glioblastoma tissue samples and cell lines [41]. Another analysis of 111 patients with glioblastoma showed that 76.6% of the specimens had a positive staining for phosphorylated STAT3-Tyr^7^^05^ (pSTAT3^Tyr7^^05^), and those patients carrying tumors with a strong staining had a shorter survival caused by a more aggressive subtype [42]. In 2008, Brantley* et al.* suggested that STAT3 may be an attractive therapeutic target for glioblastoma because STAT3 is constitutively active as a consequence of phosphorylation at both Tyr^7^^05^, as well as Ser^727^ [43]. Interestingly,* in vitro*, levels of constitutive STAT3 activation vary in cultured glioblastoma cell lines, but most cells show a constitutive expression of the IL-6 cytokine family [43]. Furthermore, the authors observed that PIAS3, an endogenous inhibitor of STAT3, was largely absent in human glioblastoma tissues. This is in contrast to SOCS3, another endogenous inhibitor of STAT3, which is a direct transcriptional target of STAT3 and highly expressed in primary tumors from glioblastoma multiforme patients [44]. Importantly, SOCS3 expression correlated positively with radioresistance in U87 cells [44]. Zhou* et al.* also demonstrated that ectopic expression of a dominant-negative variant of STAT3 (DN-STAT3-Y705F) in U87 cells resulted in a reduced survival fraction (SF) after a single irradiation with 4 Gy from 43% in control cells to 22% in DN-STAT3-Y705F transfected cells [44], indicating an increased sensitivity to irradiation. Radiosensitization after STAT3 modulation has also been shown using RNAi-based approaches in U87 and U251 glioblastoma cells* in vitro* and* in vivo* [45]. This sensitizing effect was correlated with an induction of apoptosis by the initiator caspase-8/9 and the effector caspase-3/7 [45]. Subsequently, the authors combined RNAi against STAT3 and ErbB2 with irradiation in an U251 xenograft mouse model, which led to a highly significant inhibition of tumor growth compared to single RNAi treatment without toxic effects in normal astrocytes from the brain of Wistar rats [45]. A recent study reported an increased radiosensitivity in constitutively STAT3 expressing glioblastoma multiforme cell line-derived CD133+ cells and GBM-CD133+ xenografts following treatment with the STAT3-inhibitor AG490 and resveratrol (RV) [46]. In contrast to the findings described above, two other constitutively STAT3 expressing glioblastoma cell lines (SF763 and SNB19) could not be sensitized to irradiation after treatment with JSI-124 (cucurbitacin I) or a neutralizing gp130-blocking antibody [47]. However, this conflicting observation may be explained by the fact that Chautard* et al.* used a relatively low concentration (0.01 µM), which actually failed to elicit a detectable reduction of pSTAT3^Tyr705^ by Western blot analyses.

### 2.2. Breast Cancer

Breast cancer represents the most common cancer in females worldwide and the second-leading cause of cancer-related death in the U.S. [39,48]. After breast-conserving surgery for invasive disease, adjuvant breast irradiation is recommended for most patients in order to decrease the risk of local recurrence [49,50].

Constitutive activation of STAT3 in breast cancer cell lines and primary cancers has been demonstrated by many groups [51,52,53,54]. This activation was successfully inhibited using AG490, a JAK2 inhibitor and via stable expression of a dominant negative variant of STAT3, STAT3β [55]. This led to a suppression of tumor cell growth, a decreased colony formation and an induction of apoptosis, but spared normal cells or cancer cells lacking constitutively active STAT3 [55]. Kim and colleagues were the first to demonstrate that overexpression of another dominant-negative STAT3, which inhibits phosphorylation of STAT3 at Tyr^7^^05^, resulted in radiosensitization of human MDA-MB-231 breast cancer cells [56]. Moreover, Kang* et al.* showed that treatment with the natural flavonoid compound, xanthohumol, which decreases expression levels of STAT3 and epidermal growth factor receptor (EGFR), increased the sensitivity of MCF-7 human breast cancer cells, as well as doxorubicin-resistant MCF-7 cells by inducing apoptosis [57].

### 2.3. Colorectal Cancer

Colorectal cancer (CRC) is the second leading cause of cancer-related death in the world, with more than one million new cases every year [39,58]. For locally advanced rectal cancer, the standard treatment consists of preoperative CRT, followed by radical surgical resection and postoperative chemotherapy [58,59,60].

Spitzner and colleagues were the first to demonstrate that STAT3 represents a potential molecular target for CRT-sensitization in this disease. After having established an* in vitro* model for chemoradiosensitivity, the authors showed that *STAT3* gene expression correlated positively with increasing resistance of CRC cell lines to 3 µM of 5-fluorouracil (5-FU) and 2 Gy of radiation [61]. This was subsequently confirmed on the protein level, as well [62]. While STAT3 was not constitutively active, stimulation with IL-6 resulted in remarkably higher expression levels of phosphorylated STAT3 in CRT-resistant cell lines. The authors further demonstrated that siRNA- and shRNA-mediated silencing of *STAT3*, as well as treatment with the small-molecule inhibitor STATTIC, which blocks STAT3 phosphorylation, significantly decreased clonogenic survival of CRC cell lines following exposure to 3 µM of 5-FU and irradiation in a dose-dependent manner, with dose-modifying factors (DMF) of 1.3 to 2.5 at a surviving fraction of 0.37 [62]. In addition, STATTIC-treatment resulted in CRT-sensitization in a subcutaneous xenograft model, with a significantly delayed tumor re-growth in STATTIC-treated mice compared with control animals. Additional indirect evidence that STAT3 mediates resistance to CRT comes from studies by Urick and colleagues, who treated CRC cell lines with the MEK inhibitor, selumetinib, which resulted in increased sensitivity to 5-FU-based CRT* in vitro* and* in vivo*, with DMFs at 10% survival rate of 1.52 and 1.78, respectively [63]. This CRT-sensitization was accompanied by decreased STAT3 phosphorylation and an increase in mitotic catastrophe and apoptosis.

### 2.4. Esophageal Cancer

Esophageal cancer represents another leading cause of cancer-related morbidity and mortality, with overall five-year survival rates in the range of 15% to 25% worldwide [39,64]. In locally advanced stages of the disease, treatment generally involves a multidisciplinary approach, depending on the underlying histology (squamous-cell carcinoma (SCC) or adenocarcinoma (AC)) and factors, such as localization of the tumor and comorbidities of the patient: either preoperative CRT or chemotherapy combined with radical surgical resection, or definitive CRT without surgical resection [64,65].

Chen and colleagues demonstrated that 47% of 173 SCC of the esophagus were positive for p-STAT3 in an immunohistochemical (IHC) staining, and approximately 50% were positive for pSTAT3 in a tissue microarray (TMA) [66]. Subsequently, the authors analyzed IL-6 levels in their cohort of 173 patients [67]. IL-6 was overexpressed in tumor tissues compared to adjacent non-malignant epithelial tissues, and this expression was significantly associated with poorer treatment response rates, shorter survival, and the development of distant metastases. In CE81T cells, shRNA-mediated down-regulation of IL-6 expression resulted in a significantly inhibited tumor growth* in vitro* and *in vivo*, attenuated invasive capacity of esophageal cancer cells in migration scratch assays, increased radiation sensitivity, and delayed tumor growth in mice, associated with enhanced DNA damage and reduced STAT3 activation [67].

### 2.5. Prostate Cancer

Prostate cancer represents the most common cancer type in men [39]. The management of low-risk or favorable-risk prostate cancer is highly controversial, and thus, different options, like watchful waiting, radical prostatectomy (RP) or external-beam radiation therapy (EBRT), are individually used for early stage disease [68,69]. For high-risk or locally advanced patients, there is also an ongoing discussion on the optimal multimodal management strategy. Single-modality treatment with RP or EBRT results in a similar progression-free survival of about 50% at eight years. However, multimodal approaches, including RP, EBRT and androgen-deprivation therapy (ADT), achieved the best overall long-term outcomes [70].

An analysis of 45 prostate adenocarcinomas revealed elevated STAT3 DNA-binding activity in 37 (82%) of 45 tumors when compared to matched non-carcinoma tissues [71]. Furthermore, higher levels of pStat3^Tyr7^^05^ were detected in the nuclei of epithelial tumor cells, and pSTAT3 activation was correlated significantly with more aggressive histology (Gleason scores ≥7). *In vitro*, STAT3 DNA-binding activity and constitutively active STAT3 were observed in three human prostate cancer cell lines (DU145, PC3, and LNCaP). Blockade of STAT3 activity using siRNAs resulted in reduced cell growth and increased apoptosis measured by caspase-3 activation [71]. Very recently, Wu and colleagues investigated the radiation response after manipulation of IL-6 signaling* in vitro* and* in vivo*. IL-6 inhibition enhanced the radiation sensitivity of prostate cancer cells due to increased expression of p53, increased cell death, and augmented DNA damage [31]. Furthermore, IL-6 inhibition in tumor-bearing mice resulted in decreased tumor regrowth following irradiation.

### 2.6. Bladder Cancer

Preoperative chemotherapy followed by cystectomy or CRT is commonly used to treat muscle-invasive bladder cancer. However, many tumors are resistant against multimodal treatment procedures, and unfortunately, around 50% of the patients are diagnosed with local relapse or distant metastases within five years [72,73].

In a panel of high-grade human non-muscle-invasive and muscle-invasive bladder tumors, Sun* et al.* examined cytoplasmic and nuclear staining for total and phosphorylated STAT3. Differences between tumor and normal cells were detected in nuclear stainings of total STAT3, with higher expression levels in carcinoma tissues [74]. High protein levels of phosphorylated STAT3 were only observed in tumor tissues and also in six different bladder cancer cell lines [74], indicating pronounced STAT3 activation [75]. Importantly, treatment with diindolylmethane (DIM) induced apoptosis in bladder cancer cell lines that were resistant to radiotherapy [74].

### 2.7. Cervical Cancer

Cervical cancer is the second most common tumor among women worldwide and belongs to the group of human papilloma virus (HPV)-associated cancers. It is treated with surgery or multimodality therapy and associated with a good prognosis if diagnosed in early stages of the disease [76]. For locally advanced cervical cancer, different randomized clinical trials showed a 30% to 50% decrease in risk of death by the addition of concurrent platinum-based CRT when compared with radiotherapy alone [77].

Constitutive activation of STAT3 plays a prominent role in the development and progression of cervical cancer [78]. Chen and colleagues reported constitutive activation of STAT3 in 22% of 165 cervical cancers [79]. However, no significant correlation was found with clinical parameters, like overall survival (OS), local relapse-free survival, and metastasis-free survival [79]. *In vitro* studies of human HeLa cells, which were treated with cepharanthine (CEP), established this compound as a potential radiosensitizer for cell lines with an overexpression and constitutive activation of STAT3 [80]. Both* in vivo* and* in vitro* experiments showed that the reduction of both cellular and tumor growth was more pronounced when ionizing radiation and CEP were combined (DMF = 1.31), compared with either CEP or irradiation alone [80].

### 2.8. Head and Neck Cancer

An extensive body of evidence highlighting the role of STAT3 in mediating resistance to (chemo-) radiotherapy first originated from analyses of head and neck squamous cell carcinomas (HNSCC). In Europe, the standard therapy of HNSCC, which include cancers of the oral cavity, oropharynx, hypopharynx, pharynx and larynx, consists of surgery and, in cases of locally advanced tumors, postoperative (chemo-) radiotherapy. For primary unresectable tumors, a definitive platinum-based CRT is applied [81,82,83].

STAT3 represents a particular promising target in this tumor entity, because, especially for patients with recurrent or metastatic disease, RT can be combined with an EGFR inhibitor, as activation of EGFR signaling commonly stimulates STAT3 phosphorylation [84,85]. In HNSCC, EGFR and STAT3 are overexpressed in the predominant majority of cases [86,87,88], and pSTAT3 expression correlated with poor prognosis [89,90,91]. Kruser and colleagues incubated the HNSCC cell line UM-SCC-1 with the anti-EGFR antibody panitumumab, followed by irradiation. Phosphorylation of STAT3 was inhibited by panitumumab, accompanied by a sensitization to irradiation both* in vitro* and* in vivo* [92]. Chen* et al.* used IL-6 to stimulate STAT3 signaling in the cetuximab-resistant hypopharyngeal cancer cell line FaDu (FaDu-C225-R), which resulted in an enhanced surviving fraction following irradiation when compared to unstimulated cells [90]. When IL-6 signaling was blocked by an IL-6 antibody, FaDu-C225-R cells were sensitized to radiation therapy comparable to the level of wild-type FaDu cells [90].

As shown by several groups, direct targeting of STAT3 via siRNA [93], shRNA [32,94], or the small-molecule inhibitor STATTIC [95,96] resulted in a significant radiosensitization of HNSCC cells* in vitro* and* in vivo*. Chen and colleagues demonstrated that cucurbitacin I (JSI-124), which is supposed to represent a potential chemo- and radio-sensitizer [97], decreased the expression level of pSTAT3^Tyr7^^05^ in a subset of CD44^+^/ALDH1^+^ cells from HNSCC patient-derived tumors, which showed high levels of p-STAT3 and displayed typical properties of putative cancer stem cell (CSC) [91]. This was associated with enhanced radiosensitivity, attenuated invasion capability, soft agar sphere formation, suppressed tumor growth of xenotransplanted CD44^+^/ALDH1^+^ cells, and decreased lung metastatic ability* in vivo*. Hsu and colleagues treated the radio-resistant HNSCC cell lines SCC-22A and SCC-22B, which both express constitutively activated STAT3 at high levels, with the multi-receptor tyrosine kinase inhibitor linifanib (ABT-869). Treatment with linifanib, which indirectly inhibits STAT3 phosphorylation through modulation of its upstream kinases, resulted in re-sensitization to radiation therapy [98].

Mechanistically, there is evidence to suggest that the sensitization effect following combined STAT3 inhibition and radiation in HNSCC is mediated by apoptosis [32,92,93,94,95,98], enhanced residual DNA-damage assessed by γH2AX staining [92] or comet assay [94], G2/M cell cycle arrest [98], and reduced tumor angiogenesis in murine xenografts of HNSCC cells [32].

### 2.9. Lung Cancer

Lung cancer represents the leading cause of cancer-related death worldwide in both men and women [39]. Approximately 80% of all pulmonary malignancies are non-small-cell lung cancers (NSCLC) [3]. In patients with stage II and IIIa NSCLC, a platinum-based postoperative chemotherapy has improved the long-term survival after surgery. For patients with locally advanced disease, a CRT, preferably given concurrently, is the standard approach, while a platinum-based chemotherapy has been shown to improve survival in patients with metastatic non-small-cell lung cancer [3].

From 303 cases of human NSCLC, expression of total STAT3 could be detected in 91% of cases, while pSTAT3 was present in 61% of cases [99]. These findings provide a basis for therapeutic targeting of STAT3 in this disease. Purnell* et al.* used the Src inhibitor AZD0530 to treat A549 and Calu-6 lung cancer cell lines, which resulted in a significant inhibition of cell migration, invasion and enhanced sensitivity to irradiation [100]. Treatment of HCC2429 and H460 lung cancer cells with the JAK2 inhibitor TG101209 led to a reduced phosphorylation of STAT3, inhibition of survivin, increased apoptosis, and decreased cell proliferation [101]. This resulted in enhanced radiosensitivity* in vitro*, extended tumor growth delay and a lower vascular density in lung cancer xenografts.

Another serine/threonine protein kinase that phosphorylates STAT3 is CK2. Treatment of A549 and H460 (EGFR wild-type) and H1650 and H1975 (EGFR mutant) lung cancer cell lines with three different CK2 inhibitors (4,5,6,7-tetrabromo-1H-benzotriazole (TBB), tetrabromocinnamic acid (TBCA), and hematein) resulted in reduced STAT3 activation, growth suppression, and enhanced radiosensitivity [102]. Although EGFR mutant cell lines needed higher concentrations of the CK2 inhibitors to suppress pSTAT3, successful inhibition followed by radiation resulted in radiosensitization, independent of the EGFR mutation status of the respective cell line.

Using RNAi-mediated inhibition of STAT3 expression, Yin and colleagues showed enhanced sensitization to radiotherapy in A549 and SK-MES-1 lung cancer cells [103]. This effect resulted from suppressed proliferation and increased apoptosis in shSTAT3 transfected cells and sensitization was validated in an* in vivo* mouse model. Hsu and colleagues isolated cancer stem-like CD133^+^ cells from NSCLC patients and investigated the combined effect of cucurbitacin I and irradiation [104]. They observed an inhibitor-dependent decrease of STAT3 phosphorylation, which provoked abrogated tumor growth, sphere formation ability, and increased radio- and chemo-sensitivity (cisplatin, doxorubicin, paclitaxel) in CD133^+^ NSCLC cells.

### 2.10. Skin Cancer

DNA is a strong absorber of ultraviolet B (UVB) radiation in cells, which causes DNA damage and mutations [105]. For protection from cancer, the human skin is very well adapted to this environmental stress, so that UVB-irradiated keratinocytes undergo G1 cell cycle arrest to allow for DNA damage repair or, if damage levels are too high, induce apoptotic pathways [105]. STAT3 is a key regulator of keratinocytes in response to UVB irradiation and plays an important role in initiation, promotion and progression of skin cancer [106,107,108]. Sano* et al.* demonstrated that STAT3 was constitutively activated in UVB-induced human skin cancer specimens and in a mouse model of skin cancer. Murine STAT3^−/−^ keratinocytes showed an increased UVB radiation sensitivity when compared to wild-type mice [109]. After transfer of ectopic *STAT3* plasmid DNA into STAT3-deficient mice and subsequent UVB irradiation, this sensitivity could be reversed, with concomitant reduction in UBV-induced apoptosis.

Melanoma represents the most aggressive type of skin cancer, known to be relatively resistant to radio- or chemo-therapy and with a high risk to metastasize [110]. Johnson and colleagues treated the murine melanoma cell line SW1, which is highly radioresistant, with a combination of radiation and resveratrol (RV), an inhibitor of STAT3-dependent transcription. This treatment resulted in a pronounced decrease of the clonogenic survival due to RV-induced apoptosis [111]. Similar results could be confirmed in the human melanoma cell line WM35.

Bonner and colleagues demonstrated a potential role for STAT3 signaling in mediating resistance of epithelial squamous cell carcinoma to CRT [112]. They could show that RNAi-mediated silencing of *STAT3* resulted in radiosensitization of A431 cells.

### 2.11. Other Cancers

For various tumor entities, only limited data are available. A selection of these reports is summarized below.

B-1 cells, a subset of B-cells that predominate in the peritoneal cavity and that express a constitutively active form of pSTAT3^Ser727^, are known to be resistant to irradiation [113]. Otero and colleagues investigated radiation-induced apoptosis in mouse B-1 wild-type and STAT3^−/−^ B-1 cells [25]. Whereas 80% of wild-type B-1 cells could be recovered from the peritoneal cavity 48 h after irradiation at 5 Gy, only 30% of B-1 cells lacking STAT3 survived the radiation, primarily caused by an increased apoptosis [25].

Anaplastic thyroid cancer (ATC) is a very rare, but highly dedifferentiated, tumor with a devastating prognosis, and treatment concepts typically involve a combination of surgery, radiotherapy, and chemotherapy [114]. CD133^+^ cells derived from either ATC cell lines or ATC patients have been reported to exhibit cancer stem cell properties [115]. A literature-based network analysis by Tseng* et al.* suggested that STAT3 signaling is a key factor in regulating cancer-related biomolecular signatures and pathways in ATC-CD133^+^ cells that are strongly associated with dedifferentiation and thyroid tumorigenesis [116]. Subsequently, patient-derived ATC-CD133^+^ cells were treated with the STAT3 inhibitor cucurbitacin I, which resulted in an increased sensitivity to chemotherapeutic drugs (cisplatin, 5-FU, and doxorubicin), and an increased sensitivity to different doses of ionizing radiation. In ATC-CD133^+^ xenotransplanted mice, radiation at 4 Gy combined with cisplatin-based chemotherapy therapy or cucurbitacin I alone resulted in a suppressed proliferation of CD133^+^ cells. Moreover, a combination of all three components dramatically diminished tumor growth, effectively reduced the number of lung metastases, and significantly prolonged survival when compared to single treated mice [116].

Very recently, treatment with the multiple kinase inhibitor sorafenib resulted in increased radiosensitivity of four hepatocellular carcinoma (HCC) cell lines [117]. The same result was observed with siRNAs targeting *STAT3*. Sensitizing effects were due to increased apoptosis as a result of down-regulated pSTAT3^Tyr7^^05^ and diminished activation of STAT3 signaling related proteins. To reverse the sorafenib-associated radiosensitization, Huang* et al.* generated a stable STAT3 overexpressing HCC cell line, which resulted in a complete abolishment of the previous observed sensitizing effect [117]. These results were further validated* in vivo* [117].

## 3. Potential Mechanisms of STAT3-Mediated Radio- and Chemoradiotherapy-Resistance

Because of the numerous and fairly diverse biochemical processes that STAT3 affects, it is very likely that STAT3 serves as a sensitizer to radiation and chemotherapy in more than one specific way and that it may act depending on the respective cellular and developmental context. Much of our knowledge leading to the emerging picture of the likely mechanism(s) of therapy resistance has come from studies of downstream targets in cell lines and tumor models and from* in vitro* investigations of its cellular trafficking and processing.

Evaluation of the direct targets transcriptionally activated by STAT3 has given further support to its important roles in cell growth and survival pathways. STAT3 suppresses apoptosis genes and induces expression of proliferation genes, as well as stemness-promoting or -preserving gene targets and, also, some known oncogenes [20,118,119,120,121]. Furthermore, stress response pathway components have both been found to be regulators of STAT3 activity or to be regulated by it. Several DNA repair genes are also targets of STAT3 [122,123].

Nuclear translocation of STAT3, phosphorylated as a consequence of extracellular signals received by cellular receptors, is required for any of its downstream actions. The process is not fully understood, but is known to depend on several importins [124,125,126]. Re-translocation into the cytoplasm is probably required for maintaining signal responsiveness. While nuclear export mechanisms appear to differ for different STAT proteins, nuclear export signals located in or near the DNA binding domains are critical in all cases. Elegant studies with nuclear export blockers have shown that STAT3 export is dependent on multiple NES elements. This may well be due to the fact that the essential and multiple roles of this factor necessitate especially tight regulation of its actions [127].

Of obvious clinical relevance are the actions of STAT3 in promoting cell migration and tissue invasion. Direct pharmacological inhibition of STAT3 has been shown to inhibit these processes in various settings, in some cases along with reducing the induction of the expression of known metastasis genes [74,128,129,130,131]. Moreover, STAT3 has been shown to mediate epithelial-to-mesenchymal transition (EMT) in different carcinomas, either alone or in combination with other triggers [130,132,133]. Interestingly, oxidative stress appears to impair several regulatory pathway chains to promote persistent STAT3 activation, in mesenchymal and other cell types [134].

In this context, it should also be mentioned that, in addition to the well-established pathway centering on receptor-dependent tyrosine phosphorylation of STAT3, physiological functions of STAT3 outside the nucleus, in the cytoplasm, and especially within mitochondria have been recently reported. Exploration of these non-genomic functions is still in its early stages, but important consequences for the growth and metabolism of both normal and malignant cells have been postulated [135,136]. STAT3 (and, in fact, other STATs) localizes to the mitochondria and affects the respiratory electron transport chain, adding to its roles in cellular defense against stress or injury [137,138], and reduced STAT3 expression can impair mitochondrial function [139]. Consistent with this, blocking STAT3 activity with antioxidants, as well as STAT3 activation by reactive oxygen species has been shown to enhance cell survival [140], while mitochondrial overexpression of STAT3 promoted the growth of cancer cells [141]. Targeting mitochondrial STAT3, e.g., by inhibiting its mitochondrial import, has shown promise in the treatment of pancreatic cancer in animal models [142].

As mentioned above, because of the numerous physiological processes that STAT3 affects and the large number of different signals it transduces, it is very likely that it can account for therapy resistance in more than a single way and that it may act differently depending on the cellular and developmental context.

## 4. STAT3 Inhibitors in Clinical Translation

In addition to the many biochemical and physiological observations outlined above, the idea to target JAK/STAT signaling as a therapeutic strategy to overcome resistance to chemoradiotherapy is, in large part, also inspired by the fact that a sizeable and continuously increasing number of drugs that inhibit JAK/STAT signaling have already been tested in clinical trials or have been approved by the FDA.

In the past decade, various strategies have been explored to develop and implement effective STAT3 inhibitors [143,144,145]. Substances that block STAT3 signaling were primarily identified from screenings of libraries of natural compounds or chemically synthesized small molecules [146]. For inhibitor design, different strategies are available to disrupt STAT3 activity: first, a blockade of upstream signaling receptors; second, induction of the activity of phosphatases that dephosphorylate STAT3; third, prevention of nuclear translocation and DNA binding of STAT3; and fourth, direct inhibition of the STAT3 protein via its SH2 domain or phosphorylation sites [144,145]. However, to our knowledge, none of those direct STAT3 inhibitors (several of which were successfully tested in pre-clinical research) are currently in translational clinical studies for the treatment of cancer patients. Some of the main reasons for this disconnect are the often high concentrations needed to exert their effects, poor drug delivery, toxicity* in vivo*, and stability problems due to biologically labile groups that may react with multiple targets [86,145,147].

In clear contrast, several Janus kinase (JAK) inhibitors that block upstream signaling are in clinical development. Selected orally available and adenosine triphosphate (ATP)-competitive small-molecule JAK kinase inhibitors (ruxolitinib (INC424), SAR302503 (TG101348), pacritinib (SB1518), CYT387, AZD1480, tasocitinib, INCB028050, CEP-701, XL019) have already been tested in clinical trials for myelofibrosis and rheumatoid arthritis, for pancreatic and breast cancer, and for hematologic malignancies [11,148,149,150]. Small-molecule inhibitors that interfere with STAT3 upstream signaling have also been developed for Src kinase. For instance, saracatinib (AZD0530) was tested in phase II trials for advanced non-small-cell lung cancer [151]. The dual Src/Abl inhibitor dasatinib (BMS-354825) has shown preclinical antitumor activity in solid tumors and clinical activity in leukemia [152]. Because STAT3 can also be phosphorylated by the IL-6 signaling cascade and because IL-6 is one of the most ubiquitously deregulated cytokines in cancer, it is a rational biological target for therapeutic investigations [150]. Antibodies against IL-6 (CNTO 328, siltuximab) or the IL-6 receptor (tocilizumab, REGN-88) have been used in several clinical trials in different tumor entities, including multiple myeloma, ovarian cancer, prostate cancer, and renal cell carcinoma, and have been found to be well tolerated [150,153]. Although their exact mechanism of STAT3 inhibition is not clear, two other compounds are actually tested in early phase clinical trials for cancer patients. Pyrimethamine [146], an antimalarial drug, is in phase I/II trials for the treatment of chronic lymphocytic leukemia and small lymphocytic lymphoma [154]. The NF-κB/STAT3 inhibitor RTA 402 is currently undergoing phase I/II clinical testing for pancreatic cancer [155] and a phase I trial in patients with solid tumors and lymphoid malignancies has already been completed [156].

Obviously, as JAK/STAT signaling controls a variety of cellular processes and affects the microenvironment, as well as the immune system, therapeutic targeting may have a wide range of systemic ramifications [11,145,150,157]. For instance, chronic exposure to JAK inhibitors can result in hematologic toxicities, such as anemia and thrombocytopenia, and gastrointestinal disturbances [148,149]. Pertinent to the latter, very recent evidence indicates that STAT3 inhibition may impair the host protection from intestinal bacterial infection and increase the risk for pathogen-mediated diarrhea [158]. However, while STAT3 has been shown to be essential for the early development of mouse embryos [159], the role of STAT3 in transformed cells seems to be mechanistically distinct from its function in normal cells [160], and therapeutic targeting would generally not completely abolish STAT3 expression. Furthermore, if JAK/STAT inhibitors were used as CRT-sensitizer, exposure time would be limited to a few weeks, thereby potentially avoiding the side effects of chronic inhibitor treatment. Potentially, this may even allow the application of higher inhibitor doses.

## 5. Perspective and Conclusions

Treatment resistance represents a substantial and complex problem in modern cancer therapy as more and more patients are treated with CRT or combinations of irradiation and other modalities. It can be speculated that overcoming resistance will not solely depend on the addition of conventional or new chemotherapeutic drugs, but rather will require the integration of drugs that target pathways or genetic conditions that are specifically activated or deregulated in resistant cancer cells. There is now a convincing body of evidence that JAK/STAT signaling is one such pathway that holds great promise for future treatment concepts for two main reasons:

First, STAT3 signaling is frequently activated, often constitutively, in a variety of human malignancies, including cancers of the head and neck, colorectum, cervix, breast, and esophagus. Because radiation therapy is an integral part of the respective treatment regimens, targeting STAT3 represents a promising strategy for these entities. Second, both* in vitro* and* in vivo* data convincingly suggest that JAK/STAT signaling mediates resistance to CRT and that pathway inhibition increases treatment sensitivity (Table 1). Third, an increasing number of drugs that target JAK/STAT signaling are emerging on the horizon, which are being tested in clinical trials or that have been approved by the FDA, and consequently have already been integrated into the management of patients [148,149]. Obviously, this represents an essential prerequisite for the successful transfer into clinical treatment strategies.

**Figure 2:**
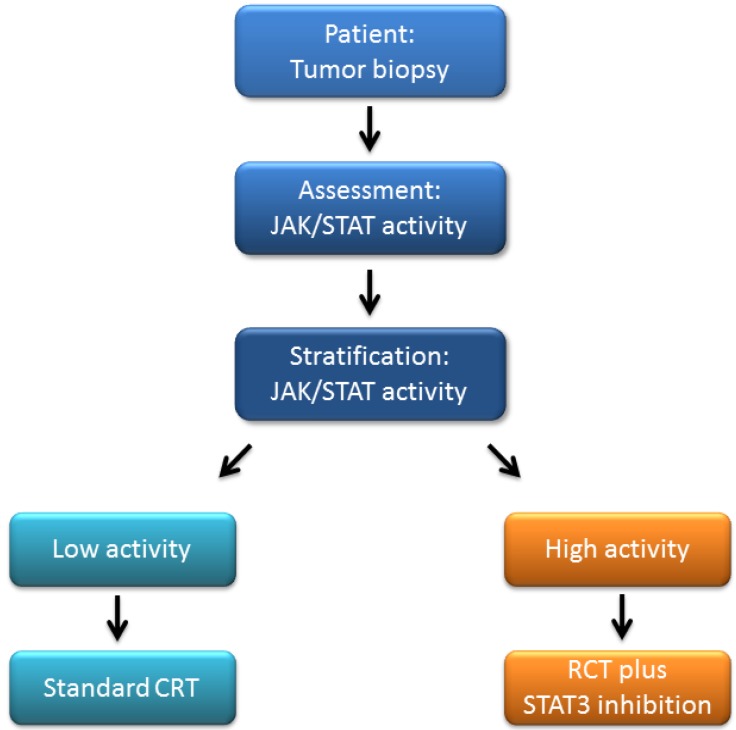
Outlook: JAK/STAT activity-guided patient stratification and management.

Thus, we envision that pre-therapeutic assessment of JAK/STAT signaling activity may help to selectively stratify patients and that CRT combined with simultaneous inhibition of JAK/STAT signaling may be implemented for tumors with high pathway activity (Figure 2). This would have considerable clinical implications.

**Table 1:** Reports on RT- or CRT-sensitization by STAT3 pathway modulation.

Tumor Type	Treatment	Pathway Modulation	Sensitization *In Vitro*	Sensitization *In Vivo*	Reference
Anaplastic thyroid cancer	RT/CRT (cisplatin)	Cucurbitacin I (JSI-124)	yes	yes	[116]
Breast cancer	RT	DN-STAT3	yes	n.a.	[56]
	RT	Xanthohumol	yes	n.a.	[57]
Cervical cancer	RT	Cepharanthine	yes	yes	[80]
Colorectal cancer	CRT (5-FU)	STAT3 shRNA STAT3 siRNA STATTIC	yes yes yes	n.a. n.a. yes	[62]
	CRT (5-FU)	Selumetinib (AZD6244)	yes	yes	[63]
Esophageal cancer	RT	IL-6 shRNA	yes	yes	[66]
Glioblastoma	RT	DN-STAT3	yes	n.a.	[44]
	RT	STAT3 siRNA	yes	yes	[45]
	RT	Resveratrol STAT3 shRNA AG490	yes yes yes	yes	[46]
	RT	Cucurbitacin I (JSI-124) gp130-blocking antibody	no no	n.a.	[47]
Head and neck cancer	RT	Panitumumab Panitumumab	yes no (SCC-1483)	yes n.a.	[92]
	RT	IL-6 antibody	yes	n.a.	[90]
	RT	STAT3 siRNA	yes	n.a.	[93]
	RT	STAT3 shRNA	n.a.	yes	[32]
	RT	STAT3 siRNA	yes	n.a.	[94]
	RT	STATTIC STATTIC	yes no (UM-SCC-22B)	yes n.a.	[95]
	RT	STATTIC	yes	n.a.	[96]
	RT	Cucurbitacin I (JSI-124)	yes	yes	[91]
	RT	Linifanib (ABT-869)	yes	n.a.	[98]
Hepatocellular carcinoma	RT	STAT3 siRNA Sorafenib	yes yes	n.a. yes	[117]
Leukemia	RT	Stat3^−/−^ mouse B-1 cells	yes	n.a.	[25]
Lung cancer	RT	Panitumumab	yes	yes	[92]
	RT	Saracatinib (AZD0530) PP2	yes no	n.a.	[100]
	RT	TG101209	yes	yes	[101]
	RT	TBB TBCA Hematein	yes yes yes	n.a.	[102]
	RT	STAT3 shRNA	yes	yes	[103]
	RT	Cucurbitacin I (JSI-124)	yes	yes	[104]
Melanoma	RT	Resveratrol	yes	n.a.	[111]
	RT	STAT3 siRNA STAT3 shRNA	yes yes	n.a.	[112]
Prostate cancer	RT	IL-6 shRNA	yes	yes	[31]
Skin cancer	UVB-RT	Stat3^−/−^ mouse keratinocytes	yes	yes	[109]

RT = radiation therapy, DN = dominant negative, n.a. = not applicable, DMF = dose modifying factor, CRT = chemoradiotherapy, 5-FU = 5-fluorouracil, IL-6 = interleukin-6, PP2 = 4-amino-5-(4-chlorophenyl)-7-(dimethylethyl)pyrazolo[3,4-d]pyrimidine, TBB = 4,5,6,7-Tetrabromo-1H-benzotriazole, TBCA = Tetrabromocinnamic acid, UVB = ultraviolet B.
